# A case series of mucormycosis mimics on MRI—Tales of respite amidst the havoc

**DOI:** 10.1186/s43163-022-00261-7

**Published:** 2022-06-13

**Authors:** Paresh Sukhani, Alka Goyal, Ashwini Bellamkondi, Kuldeep Mendiratta, Bhavyansh Rathi

**Affiliations:** 1 Department of Radiodiagnosis, Mahatma Gandhi Medical College, Jaipur, Rajasthan India; 2 Department of Radiodiagnosis, SMS Medical College and Hospital, Jaipur, Rajasthan India

**Keywords:** Mucormycosis, Magnetic resonance imaging (MRI), Paranasal sinuses (PNS), Computed tomography severity score (CTSS), Invasive fungal rhinosinusitis (IFRS)

## Abstract

**Background:**

Rhinocerebral mucormycosis is new bandit amidst present COVID-19 pandemic, it is an acute and lethal opportunistic fungal infection affecting immunocompromised and diabetic patients. Since the disease has got high morbidity and mortality despite aggressive treatment, radiologists play a very crucial role in early and accurate diagnosis. Erroneous diagnosis should be refrained by logistic approach and thorough clinico-radiological correlation.

**Material and methods:**

Ours was a cross sectional study included six cases after taking written informed consent who recently presented with mucormycosis like symptoms and imaging findings during a period of 1 month, but by detailed clinical and radiological evaluation, we concluded that all these cases were either physiological mimics or extraneous artefacts, this helped greatly in relieving undue anxiety of patients and referral physicians and also avoided unnecessary further workup. This study was conducted after approval by the institutional ethical committee.

**Results:**

Our study included 3 males and 3 females of age ranging from 32 to 62 years, all of which had history of COVID-positive having mild to moderate CT severity score who were treated with steroids and oxygen therapy (except one case). The most common presenting symptom was headache followed by nasal congestion. The mucor mimickers encountered were benign black turbinate sign, artifacts due to cosmetic dermal fillers and dental fillings, hemangioma, prolonged prone ventilation, and fungal ball.

**Conclusions:**

Amidst the sudden spurt in the number of cases of mucormycosis in our country in the present COVID era, there has been an increase in the number of imaging requisitions. This series of cases aims to sensitize radiologists about the importance of detailed clinical history, thorough clinic-radiological correlation and at times also taking extra efforts to reconnect to patients regarding specific clinical history and avoid fallacious diagnosis.

## Background

Mucormycosis is a fulminant, opportunistic angio-invasive fungal infection caused by class phycomycetes [1, 2]. Its prevalence in India is about 80 times that in developed countries [1].

The most common risk factors are diabetic ketoacidosis, immunosuppressant therapy, bone marrow transplantation, iron and aluminium overload and deferoxamine therapy, etc.

The mold is angiotropic and angioinvasive. The progression of the disease from nose/sinuses to deep neck spaces, orbits and cranium is either direct or through vascular invasion.

Imaging has a pivotal role in diagnosis and extent of mucor. However, we came across certain imaging mimics which were mostly incidental findings and not mucor, giving a sigh of relief.

## Material and methods

Our study aimed at studying mimics of mucormycosis encountered on MRI thereby refraining erroneous diagnosis. Ours was a cross sectional study conducted in a tertiary care hospital included six cases after taking written informed consent who recently presented with mucormycosis like symptoms and imaging findings during a period of 1 month, but by detailed clinical and radiological evaluation, we concluded that all these cases were either physiological mimics or extraneous artefacts, this helped greatly in relieving undue anxiety of patients and referral physicians and also avoided unnecessary further workup. This study was conducted after approval by the institutional ethical committee.

## Results

During 1 month period of our study 6 cases were studied for suspected invasive fungal sinusitis which included 3 (50%) males and 3 (50%) females of age ranging from 32 to 62 years (mean age 51.1 years), all of which had history of COVID-19 pneumonia having mild to moderate CT severity score (average score 13) who were treated with steroids and oxygen therapy (except case 2). The most common presenting symptom was headache (83.3%) followed by nasal congestion (66.7%). Average days of presentation after recovery from COVID-19 pneumonia was 6 days, that is, most of the patients presented during first week of recovery. The most common risk factor associated was diabetes (50%) followed by hypertension (16.7%).

The mucor mimickers encountered were benign black turbinate sign, artifacts due to cosmetic dermal fillers and dental fillings, hemangioma, prolonged prone ventilation, and fungal ball.

The radiological findings were clinically correlated.

### Findings of the study

#### Case 1

A 48-year-old male, known uncontrolled diabetic on insulin therapy tested COVID-positive 20 days back. Blood tests revealed raised CRP and IL-6 levels for which he was admitted and treated with intravenous steroids and high flow nasal oxygen. He had moderate CT severity score on HRCT chest (14/25).

**Fig. 1:**
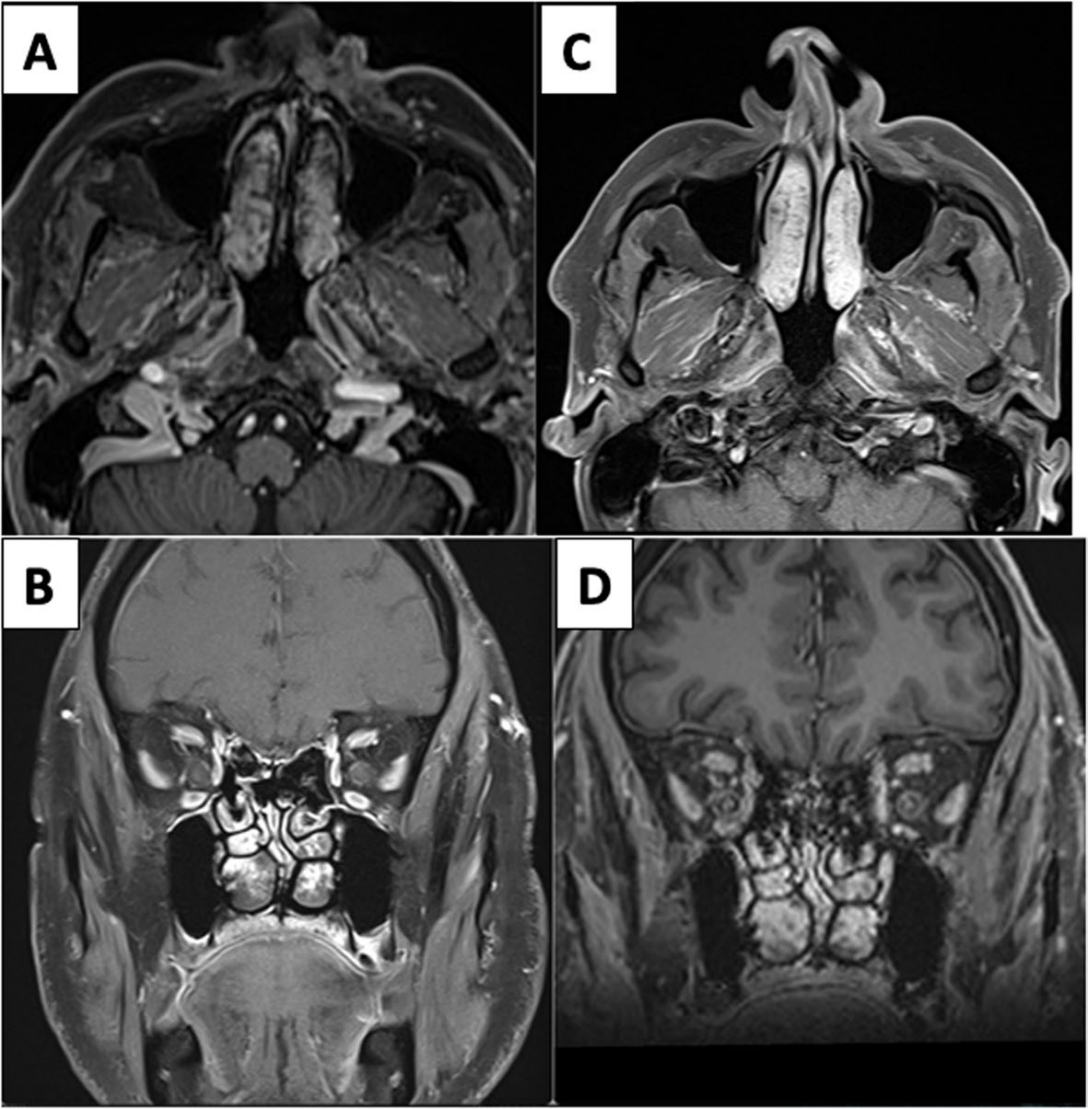
**A**, **B** Dynamic post-contrast axial and coronal scans show non-enhancing dots and striations in bilateralinferior and left middle turbinates on early dynamic post-contrast images. **C**, **D** These show progressive enhancement on delayed scans essentially ruling out pathological black turbinate

Patient complained of left hemicranial headache three days after discharge. In view of risk factors like uncontrolled diabetes, oxygen/steroid therapy, patient underwent MRI PNS: axial and coronal T2w images showed mild hypertrophy of bilateral middle and inferior turbinates. Immediate post-contrast axial followed by coronal scans showed heterogeneously enhancing left middle and bilateral inferior turbinates predominantly along posterior aspects with multiple non-enhancing dots and striations; however turbinates had sharp margins. Delayed 3D images with axial and coronal reconstruction taken approximately 6 min later showed uniform enhancement of turbinates suggesting physiological pattern of enhancement (essentially ruling out devitalized tissue) (Fig. 1).

#### Case 2

A 55-year-old female non-diabetic and hypertensive tested COVID-positive for which she was prescribed oral steroids and never required oxygen therapy. HRCT revealed moderate CT severity score of 12/25. Patient developed nasal stuffiness and headache without facial pain 5 days after testing COVID-negative.

**Fig. 2:**
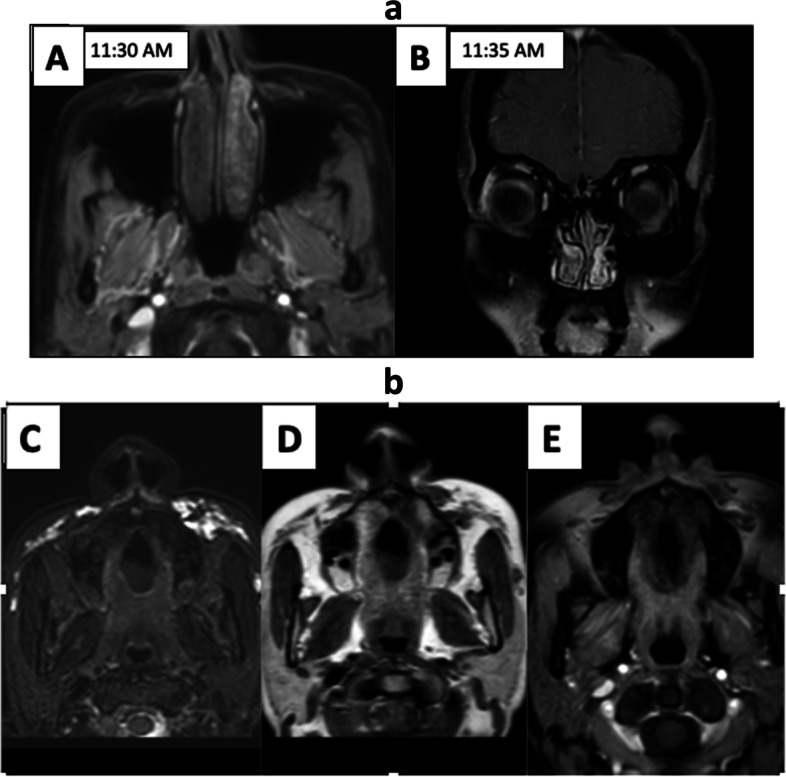
**a** (**A**, **B**) Axial T1 post-contrast image shows smooth complete rim enhancement with central non-enhancement in right inferior turbinate. Delayed scan taken 5 min later shows progressively increasing enhancement of central area essentially ruling out pathological black turbinate. **b** Axial T2 fat sat image (**C**) of this patient shows hyperintensities in subcutaneous fat involving maxillary and zygomatic region (L>R), showing hypointense signal on T1 images (**D**) and no contrast enhancement (**E**) consistent with cosmetic injection (dermal fillers)

As the patient was anxious for fungal sinusitis, MRI PNS was done which showed hyperintensities in subcutaneous fat of bilateral maxillary and zygomatic regions (L>R). To further add to confusion, early post-contrast scan showed non enhancing central portion of right inferior turbinate. Delayed scans showed progressive contrast filling in of turbinate-suggesting benign black turbinate. Subcutaneous hyperintensities did not enhance on early and delayed dynamic post-contrast images, ruling out inflammation in this region (Fig. 2a, b). On taking detailed clinical history, patient had cosmetic injection (dermal fillers) in this region which were mimicking pre malar inflammatory fat stranding (often seen in IFRS).

#### Case 3

**Fig. 3:**
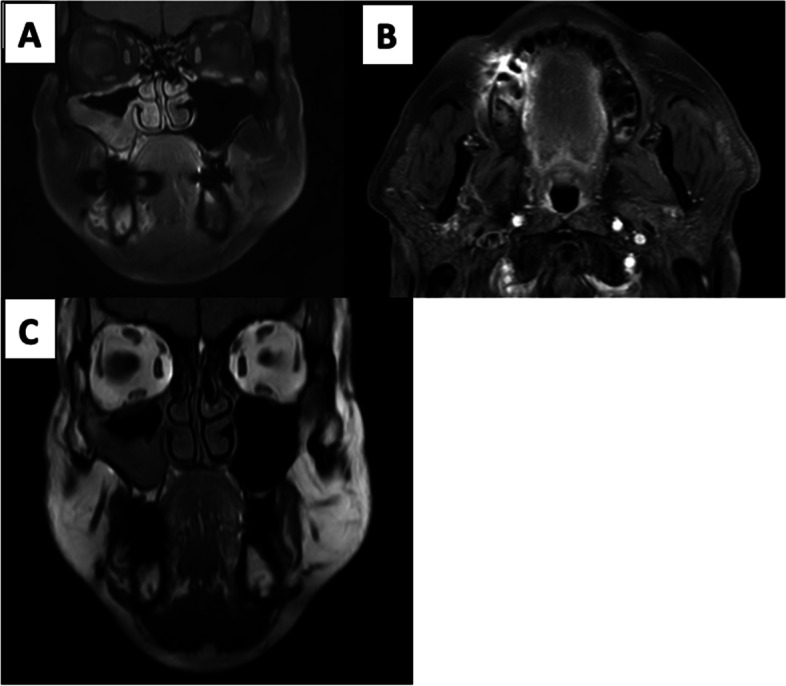
**A**, **B** Coronal and axial T1 fat-sat post-contrast images show right maxillary sinusitis and artefacts from dental fillings causing hyperintensities in right half of alveolar processes of maxilla and mandible, buccal, and lingual aspects of oral cavity. **C** Non-fat-sat coronal T1 image shows preserved normal fatty marrow signal of maxillary and alveolar processes on the right side

A 62-year-old male patient, known diabetic tested COVID-positive 2 weeks back for which he was admitted for 7 days and treated with intravenous steroids and non-invasive ventilation. Patient developed nasal congestion and right hemicranial headache 1 week after discharge. MRI PNS showed mucosal thickening of bilateral inferior and middle turbinates and right maxillary sinus. Ill-defined T2 STIR hyperintensities were seen on buccal and lingual aspects of maxillary and mandibular alveolar processes on right side. In lieu of recent hospital admission for COVID pneumonitis, uncontrolled diabetes, and treatment with IV steroids, imaging findings were suspicious for acute invasive rhinosinusitis. On post-contrast scans, normal enhancement of turbinates and right maxillary sinus mucosa was seen with no evidence of necrosis contradicting non contrast findings. In addition, coronal T1-weighted non-fat sat images were reevaluated which showed preserved normal fatty marrow of the alveolar process of maxilla, essentially ruling out marrow edema/infiltration (Fig. 3). Since non-contrast and contrast imaging findings were non-corroborative, the possibility of buccal and lingual hyperintensities being due to metallic susceptibility artefacts was kept. Physical examination of the oral cavity also revealed healthy buccal mucosa.

The reason for these artefacts was dental fillings (these were confirmed in detailed clinical history). This study highlights the importance of non-fat suppressed images, particularly in regions of buccal space and orbits.

#### Case 4

**Fig. 4:**
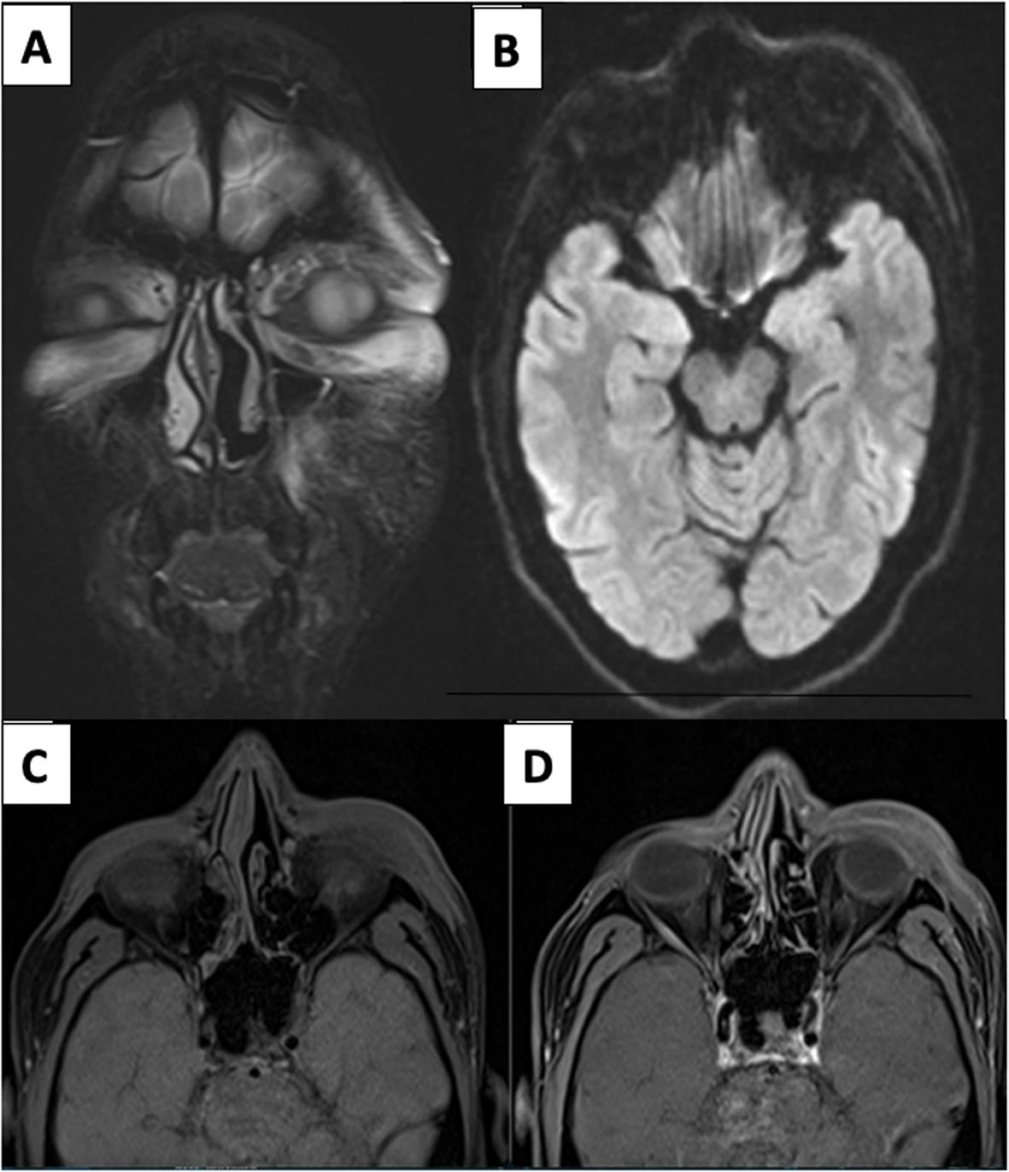
**A** Coronal fat sat T2-weighted images show bilateral (L>R) orbital hyperintensities involving preseptal, premalar fat, and frontal scalp tissue on the left side. **B** Axial DWI shows no evidence of diffusion restriction. **C**, **D** Pre- and post-contrast fat sat T1 axial images show no peripheral enhancement

A 51-year-old female, known diabetic on oral hypoglycemics, tested COVID-positive for which she was admitted for 10 days and treated with intravenous steroids and was kept on mechanical ventilation in a prone position. Biochemical tests and arterial blood gas analysis (ABG) provided information of the following: blood sugar 457 mg/dl, creatinine 1.4 mg/dl, and pH 7.2. During her hospital stay, she developed nasal congestion and periorbital edema (left>right), for which magnetic resonance imaging (MRI) of paranasal sinuses (PNS) was advised which revealed mild mucosal thickening in left maxillary and ethmoid sinuses as well as bilateral (L>R) preseptal and left pre-malar hyperintensities raising suspicion of invasive fungal sinusitis. There was no evidence of diffusion restriction in the center or periphery of this lesion with no peripheral rim of contrast enhancement. Extraocular muscles were unremarkable with no evidence of post-septal cellulitis/intracranial extension of disease process (Fig. 4).

In view of the history of prolonged prone ventilation, the patient was placed in supine position for an hour and repeat MRI PNS was done which showed significant resolution of preseptal and premalar hyperintensities suggesting dependent subcutaneous edema obviating need for further evaluation.

#### Case 5

**Fig. 5:**
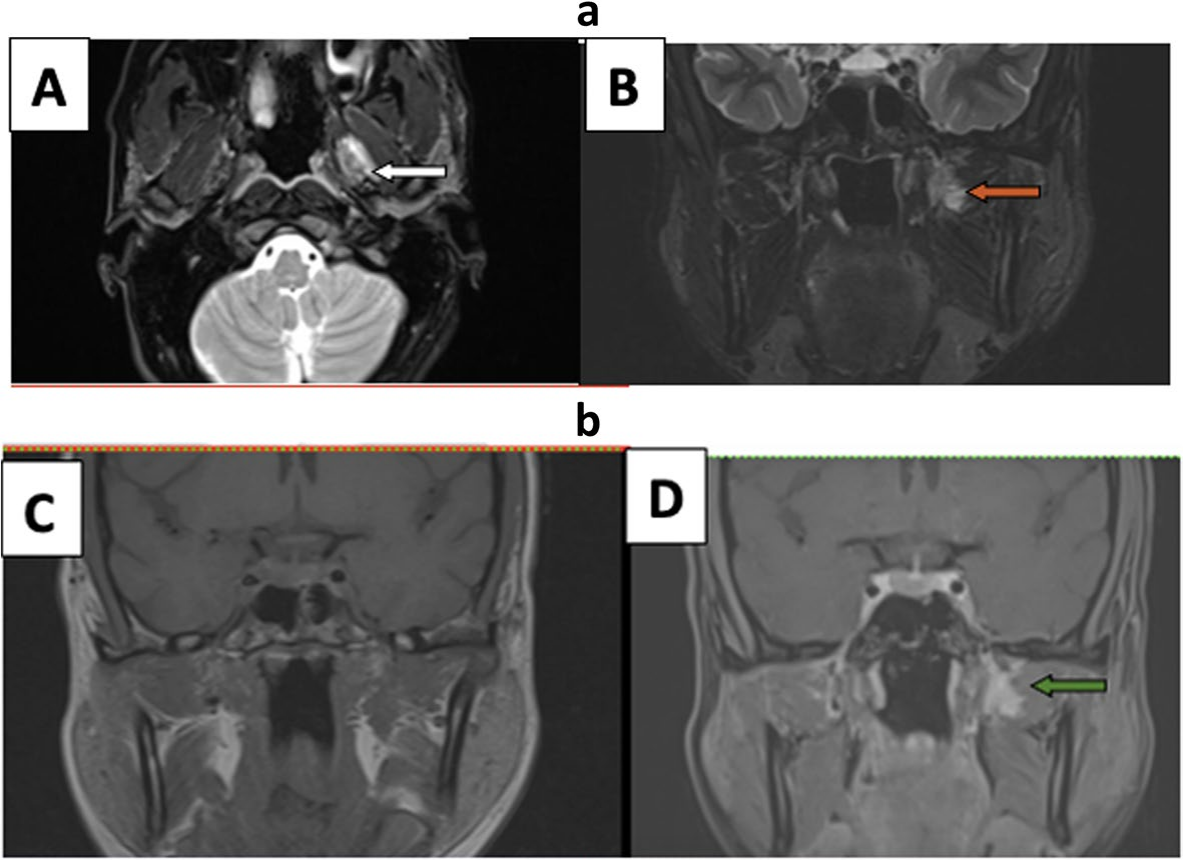
**a (A**, **B**) This patient with asymmetric ethmoidal and maxillary sinusitis (L>R) shows hyperintense lesion in left masticator space on axial and coronal T2 fat-sat images (white and red arrows), also note there is a normal area between posterior aspect of maxillary sinus and lesion with no involvement of retroantral fat. **b** (**C**, **D**) Plain and post-contrast fat sat T1-weighted images confirmed lesion to be hemangioma in view of avid enhancement (green arrow) of lesion

A 32-year-old female, non-diabetic tested COVID-positive 15 days back for which she was treated with oral steroids and intermittent oxygen therapy through nasal cannula. Biochemical and ABG values were unremarkable. Patient had a history of sinusitis and now developed increasing nasal congestion and left hemicranial headache. MRI PNS revealed T2/STIR hyperintense mucosal thickening in left maxillary sinus and bilateral ethmoid sinuses. Additionally, there was a T2/STIR hyperintense area in left masticator space adjacent to pterygoid muscles which was isointense on T1-weighted images. This raised suspicion for post-antral fat stranding. However, axial images did not show continuation of this lesion with the posterior aspect of maxillary sinus with preservation of retroantral fat. Post-contrast scans showed normal non-necrotic enhancing mucosa of above sinuses; masticator space lesion (approx.18 × 10 mm) showed avid contrast enhancement suggestive of hemangioma (Fig. 5a, b).

#### Case 6

A 59-year-old male post-COVID patient presented with complaints of swelling of left cheek, headache and left hemifacial pain. Patient had a history of hospital admission 1 week back for COVID-19 pneumonia and was treated with IV steroids and oxygen therapy. His blood sugar was uncontrolled during hospital stay and CTSS was severe (18/25). High index of suspicion for invasive fungal sinusitis led to contrast enhanced MRI PNS which revealed mucosal thickening involving both maxillary sinuses with obliteration of bilateral osteomeatal complexes and hyperintensities along left premalar space as well as adjacent maxillary alveolus suggestive of invasive fungal sinusitis.

**Fig. 6:**
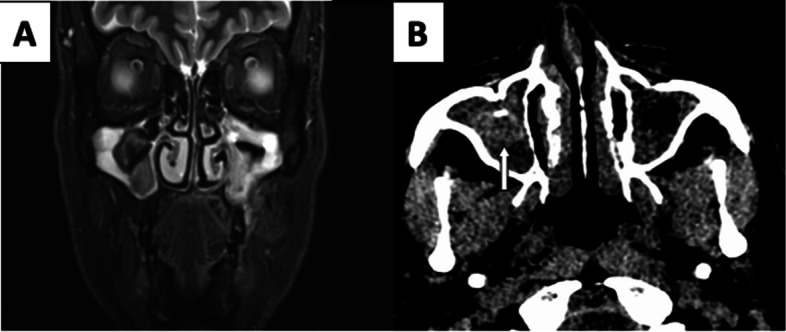
This case highlights IFRS on the left side. **A** Coronal fat sat T2W image showing involvement of left half of maxillary alveolar process and adjacent soft tissue and non-invasive fungal ball with calcification in right maxillary sinus (white solid arrow in **B**). Axial NCCT

Additionally, on the right side, there was a well-defined markedly T2 hypointense and T1 hyperintense area in the center of the right maxillary sinus without periantral inflammatory fat stranding, ruling out invasive process. In view of well-defined outlines of lesion, NCCT screening was done which showed corresponding rounded hyperdense lesion with eccentric calcification suggesting fungal ball (Fig. 6).

## Discussion

The most commonly accepted classification system classifies fungal rhinosinusitis (FRS) into invasive and non-invasive subtypes depending on evidence of tissue invasion by fungi. Invasion is defined by the presence of fungal elements within the mucosa, submucosa, bones, or blood vessels of the paranasal sinuses.

Invasive FRS is further sub-divided into acute invasive and chronic invasive categories. The chronic invasive category includes chronic granulomatous disease. Non-invasive fungal disease comprises of fungal ball and allergic fungal rhino-sinusitis [3].

Invasive disease causes tissue destruction, such that it extends beyond the bony confines of the sinuses. Duration of symptoms of acute IFRS is usually less than 4 weeks.

Major cause of mortality and morbidity in mucormycosis is due to angioinvasion leading to endarteritis and tissue necrosis which may even occur in absence of frank bony erosion.

Large areas of central non-enhancing necrotic/devitalized tissue with or without peripheral thin irregular enhancement are seen on post-contrast images in IFRS [4].

Role of a radiologist is to accurately diagnose as well as evaluate the extension which is vital for management [5]. The primary guideline for treating the disease is to reverse the underlying disease process and immunosuppression. The two mainstays of treatment are medical treatment with systemic antifungal agents, mainly intravenous liposomal amphotericin B and surgical debridement of necrosed or non-viable tissue [4, 6].

A high index of clinical and radiological suspicion is required for prompt diagnosis of IFRS, as delay in diagnosis can lead to significant increase in morbidity and mortality.

There are various non-specific imaging findings in the region of paranasal sinuses and surrounding soft tissues which mimic acute IFRS. In this article, we present a series of six cases of mimics of mucormycosis and salient points to differentiate them from true pathological findings of IFRS.

The nasal cycle is a normal physiological phenomenon in which asymmetry in blood flow leads to engorgement of erectile cavernosal tissue in predominantly inferior turbinate of one side nasal cavity over other and along anterior part of the nasal septum. This is reflected radiologically as well in form of increased nasal mucosal volume and T2-weighted signal intensities alternating on either sides of nasal cavity. Pathologically inflamed mucosa also shows similar T2 hyperintensity; therefore, awareness is important to reduce the likelihood of inflammatory disease being confused with normal physiologic changes [7].

There are various patterns of physiological enhancement of nasal turbinates due to differential distribution of cavernosal tissue in the turbinates as well as diurnal variations of nasal cycle. These include rim enhancement with well-defined borders, scattered non-enhancing foci with peripheral rim enhancement resulting in dot and striation pattern, focal non-enhancing area predominantly in the posterior turbinate with homogenous enhancement of anterior part, and lastly smooth intense homogenous enhancement [8].

Presence of sharply demarcated enhancing rim and progressive central enhancement on delayed images differentiates benign black turbinate from pathological black turbinate seen in invasive fungal sinusitis. This also highlights the importance of delayed scans taken after 5 min in suspected fungal sinusitis.

Typical rhino-nasal mucormycosis begins in the nasal cavity (predominantly middle turbinates) as the primary site of infection from where it spreads to paranasal sinuses. It presents in the early phase as non-specific mucosal thickening which is also seen in other fungal, bacterial, and viral sinusitis. Hence, CT scans are insensitive for early disease [9]. MR imaging is sensitive to pick early changes limited to nasal cavity owing to its higher spatial resolution. Most characteristic finding on MRI is black turbinate sign which results from ischemic necrosis of affected nasal turbinate and overlying mucosa leading to non-enhancement [10].

The first case in our series showed dots and striations pattern while second case showed central non enhancing mucosa with peripheral enhancing rim in early phases. Both these cases showed progressive central filling in of contrast in delayed scans. Hence, knowledge of above-mentioned physiological patterns of turbinate enhancement is important to accurately differentiate benign black turbinate from overenthusiastic diagnosis of pathological black turbinate.

MR imaging plays a pivotal role in the initial evaluation of invasive sinusitis because of its ability to better depict advanced disease involving retroantral fat, premalar fat, buccal space, orbits, deep neck spaces, and intracranial extension along the cavernous sinuses, meninges, and brain parenchyma. MRI is the only imaging modality for evaluation of perineural spread which is one of the characteristic feature of mucormycosis.

Infiltration of the periantral fat planes represents the earliest imaging finding in invasive disease, seen as ill-defined hyperintensities on fat suppressed images with heterogeneous irregular contrast enhancement in retroantral and premalar fat.

Our second case, apart from benign black turbinate sign also showed hyperintensities in subcutaneous fat along the maxillary and zygomatic regions which may mimic premalar fat stranding seen in early invasive mucormycosis. However, absence of contrast enhancement on dynamic imaging prompted us to go into detailed clinical history which revealed injection of cosmetic dermal fillers in corresponding areas.

Similar premalar and buccal space hyperintensities may be seen due to metallic susceptibility artefacts in this region. Our third case had dental fillings which produced artifactual distortion of adjacent fat planes. Detailed clinical history and careful reevaluation of coronal T1 non-fat sat images showed hyperintense normal fatty marrow of alveolar processes, further confirming these changes to be artefactual.

Orbital involvement can result in pre- and post-septal cellulitis, subperiosteal abscess, ischemic optic neuropathy, optic neuritis/perineuritis, and orbital apex involvement [11]. On MRI, pre- and post-septal orbital collections usually show peripheral contrast enhancement and diffusion restriction. Thickening and displacement of the medial rectus muscle and adjacent extraconal fat are features of orbital invasion due to weak thin lamina papyracea which forms medial wall [12].

In our fourth case, bilateral (L>R) preseptal and left pre-malar hyperintensities were seen mimicking preseptal orbital cellulitis. However, there was no evidence of diffusion restriction in the centre or periphery with no peripheral rim of contrast enhancement. Extraconal fat and medial recti were normal.

Patients in critical care settings for long duration, usually develop dependent subcutaneous edema either due to impaired fluid mobilisation accounting to negative influence on lymphatic flow of reduced muscle activity or increased central venous pressure by mechanical ventilation. Keeping this in mind, the study was repeated in supine position which showed significant resolution of preseptal/premalar abnormal hyperintensities, confirming changes of dependent edema. Prolonged prone position ventilation is an established method to improve oxygenation in the present pandemic era; hence, radiologists should be made aware of peculiar imaging findings of prolonged prone position which may mimic disease processes. In addition, this case also highlights the importance of diffusion-weighted sequence and peripheral enhancement in pathological collections.

Further extension of disease process in mucor causes involvement of the neck spaces which is characterized by obliteration of the normal fat planes in the pterygopalatine fossa, pterygomaxillary fissure, infratemporal fossa, and masticator space [13]. These changes in deep neck spaces are usually contiguous with inflammatory stranding in the retroantral region. However, there was sparing of retroantral fat in our fifth case which showed abnormal hyperintensity in left masticator space. This lesion showed avid post-contrast enhancement with no central non-enhancing areas, confirming diagnosis of hemangioma and refuting the infective process.

Intracranial findings include pachymeningitis, epidural and subdural abscesses, cavernous and sagittal sinus thrombosis, infarcts related to vascular thrombosis, mycotic emboli, and cerebral abscesses.

Acute nature of IFRS (duration < 4 weeks) leads to extensive amounts of necrosed tissue with sparse fungal hyphae. Non-invasive fungal disease like fungal ball or allergic fungal rhinosinusitis lack extensive necrosis and instead have dense fungal colonization or allergic mucin respectively, therefore appear more well-marginated and T2 hypointense.

Our last case had typical imaging findings of IFRS on the left side and fungal ball on the right side. The presence of T2 hypointense round area in right maxillary sinus without bony erosion and remodelling, prompted us to do CT screening which demonstrated characteristic eccentric calcification in corresponding hyperdense round intraluminal lesion consistent with typical fungal ball [13].

## Conclusion

Amidst the sudden spurt in the number of cases of mucormycosis in our country in the present COVID era, there has been an increase in the number of imaging requisitions. This series of cases aims to sensitize radiologists about the importance of detailed clinical history, thorough clinicoradiological correlation, and at times also taking extra efforts to reconnect to patients regarding specific clinical history and avoid fallacious diagnosis.

## Data Availability

All data generated or analyzed during this study are included in this published article.
